# Next‐generation sequencing‐based analysis to assess the pattern of relapse in patients with Philadelphia‐positive acute lymphoblastic leukemia

**DOI:** 10.1002/jha2.514

**Published:** 2022-09-09

**Authors:** Jae‐Sook Ahn, TaeHyung Kim, Sung‐Hoon Jung, Seo‐Yeon Ahn, Ga‐Young Song, Mihee Kim, Deok‐Hwan Yang, Je‐Jung Lee, Mi Yeon Kim, Joon Ho Moon, Zhaolei Zhang, Hyeoung‐Joon Kim, Dennis Dong Hwan Kim

**Affiliations:** ^1^ Department of Internal Medicine, Chonnam National University Hwasun Hospital Chonnam National University Gwangju Republic of Korea; ^2^ Genomic Research Center for Hematopoietic Diseases Chonnam National University Hwasun Hospital Jeollanam‐do Republic of Korea; ^3^ The Donnelly Centre for Cellular and Biomolecular Research University of Toronto Toronto Ontario Canada; ^4^ Department of Computer Science University of Toronto Toronto Ontario Canada; ^5^ Department of Hematology‐Oncology Kyungpook National University Hospital, School of Medicine, Kyungpook National University Daegu Republic of Korea; ^6^ Department of Medical Oncology and Hematology, Princess Margaret Cancer Centre University of Toronto Toronto Ontario Canada; ^7^ Department of Molecular Genetics University of Toronto Toronto Ontario Canada

**Keywords:** NGS, Philadelphia‐positive ALL, relapse

## Abstract

In this study, we performed serial monitoring using targeted DNA sequencing to identify genetic alterations in adults with Philadelphia‐positive acute lymphoblastic leukemia (Ph‐ALL). Deep sequencing was performed by targeting the coding regions of 45 genes with recurrent driver mutations and 1129 single nucleotide polymorphism sites. Of the 43 patients that we examined, at least one case of genetic alterations was detected in 38 (88%) of the 43 patients at diagnosis (somatic mutations in 10 patients [23%] and copy number aberrations [CNA] in 36 patients [84%]). The most frequently detected CNA lesions were in *IKZF1* (*n* = 25, 58%) and the most frequently mutated gene was *SETD2* (*n* = 5). At least one genetic abnormality (loss, gain, or persistence) was observed in all the samples obtained at relapse that were available for analysis (*n* = 15), compared with the samples obtained at diagnosis (disappearance of any previously detected genetic alterations: 11 patients [73%]; new genetic abnormalities: nine patients [60%]; and persistent genetic abnormalities: eight patients [53%]]. The most frequently deleted lesions were in *IKZF1* (*n* = 9, 60%), and the most frequently mutated gene was *ABL1* (eight patients, 53%). Our data indicate that leukemic progression may be associated with complex genetic alterations in Ph‐ALL during the course of treatment.

## 1 INTRODUCTION

The Philadelphia (Ph) chromosome, detected in 25% of adult B‐cell acute lymphoblastic leukemia (ALL), is the most common chromosomal abnormality seen in ALL [1]. Prior to the introduction of tyrosine kinase inhibitors (TKIs), the prognosis of Ph‐positive ALL (Ph‐ALL) was extremely poor, with a long‐term survival rate of less than 20% [2]. The advent of TKIs has increased the curative rates and survival outcomes of patients with Ph‐ALL. The addition of dasatinib or nilotinib to chemotherapy, followed by allogeneic hematopoietic cell transplantation (HCT), has also shown beneficial results in terms of patients becoming relapse‐free and in terms of overall survival (OS) [3, 4]. However, the most common cause of treatment failure for Ph‐ALL is relapse, despite the combined treatment of TKIs and subsequent allogeneic HCT; the outcome of patients who relapse with Ph‐positive ALL has been dismal [5, 6, 7].

Genetic heterogeneity and clonal evolution during leukemic relapse were identified using whole‐exome sequencing in ALL [8–10]. Oncogenic gene fusion and rearrangement, somatic mutation, and copy number alterations (CNAs) are evident in all cases of B‐ALL at diagnosis [8, 11]. About 80% of CNAs, or structural variations present at diagnosis, persist to relapse in B‐ALL, and 40% of CNAs and 7% of structural variations are relapse‐specific [8]. Seventy‐five percent of the relapsed tumors are descendants of minor subclones at diagnosis in B‐ALL, and several mutations are known to be responsible for resistance to specific therapeutic agents [8]. Unlike Ph‐negative ALL, Ph‐ALL is a genetically distinct subgroup characterized by unique genetic profiles at diagnosis and relapse [12, 13, 14]. *ABL1* kinase domain (KD) mutations have been reported in 50%–80% of cases in which Ph‐ALL relapsed or was refractory, which directly explains the cause of resistance to TKIs [15]. In addition, 66%–88% of patients with Ph‐ALL carry additional CNAs; the presence of specific CNAs has been shown to have dismal prognostic implications [12, 16, 17]. Genetic deletion of *IKZF1* or *CDKN 2A/B* has been reported to affect the prognosis of Ph‐ALL patients despite allogeneic HCT [12, 18–20]. *BCR‐ABL1* and *IKZF1* deletion are strongly linked and co‐occur in 70%–80% of Ph‐ALL patients, influencing the mechanism underlying drug resistance in Ph‐ALL [20, 21]; additionally, in Ph‐ALL, *IKZF1* deletions are further enriched at relapse with subclone or de novo acquisitions [22, 23].

However, since most previous findings are based on comprehensive analyses such as whole‐genome sequencing, the practical application of these results is limited due to the difficulty of in silico analysis. Therefore, a genomic analysis that encompasses these two aberrations with a clinically appropriate capability is necessary to elucidate the patterns of relapse in the Ph‐ALL subgroup. To address these genetically important issues, we performed targeted DNA sequencing designed to detect CNAs and molecular mutations to identify the diversity of genetic changes in adult Ph‐ALL patients by means of serial monitoring according to disease status.

## 2 MATERIALS AND METHODS

### 2.1 Patients

We enrolled 43 consecutive patients diagnosed with Ph‐ALL between 2003 and 2017 at a single institution. All patients received intensive induction chemotherapy including TKIs. Twenty‐nine patients received cyclophosphamide, vincristine, doxorubicin, and dexamethasone (hyper‐CVAD) induction therapy with imatinib mesylate: 600 mg for 24 of them and an additional 140 mg of dasatinib for five of them [24]. The other 14 patients were administered the daunorubicin, vincristine, and prednisolone (VPD) regimen with 600 mg of imatinib (*n* = 10) or 800 mg of nilotinib (*n* = 4) [4, 6]. Patients who had achieved complete remission (CR) morphologically received consolidation chemotherapy with or without allogeneic HCT depending on the availability of a matched, related, or unrelated donor. Transplanted patients were re‐administered TKIs in cases of relapse. Details of the treatments received are described in Table 1. All the procedures followed were in accordance with the ethical standards of the responsible committee on human experimentation (institutional and national) and with the Helsinki Declaration of 1975, as revised in 2008. Informed consent regarding the inclusion in the present study was obtained from all patients.

**TABLE 1:** Clinical characteristics of 43 patients with Philadelphia‐positive acute lymphoblastic leukemia (ALL)

	Total (N = 43)
Age in years, median (range)	46 (18–69)
Sex, male (%)	21 (54)
WBC, × 10^9^/l, median (range)	38.0 (0.8–243.2)
Performance status at diagnosis	
ECOG 1 (%)	32 (74)
ECOG 2–3 (%)	11 (26)
Type of induction therapy	
Hyper‐CVAD+ imatinib mesylate (%)	24 (56)
Hyper‐CVAD+ dasatinib (%)	5 (12)
VPD + imatinib mesylate (%)	10 (23)
VPD + nilotinib (%)	4 (9)
Achievement of CR (%)	41 (95)
Achievement of MMR after induction chemotherapy (%)	18(56*)
Achievement of CMR during chemotherapy (%)	25 (58)
No of patients received allogeneic HCT (%)	25 (63**)
Conditioning regimen (myeloablative) (%)	14 (56)
PBSC as a source of stem cells (%)	24 (96)
Donor type	
Matched‐related donor (%)	12 (48)
Unrelated donor (%)	13 (52)
T‐cell depletion as GVHD prophylaxis (%)	9 (36)
Chronic GVHD (%)	12 (48)

Abbreviations: CMR, complete molecular response; CR, complete remission; ECOG, Eastern Cooperative Oncology Group; GVHD, graft versus host disease; HCT, hematopoietic cell transplantation; Hyper‐CVAD, cyclophosphamide, vincristine, doxorubicin and dexamethasone; MMR, major molecular response; PBSC, peripheral blood stem cell; VPD, daunorubicin, vincristine, and prednisolone; WBC, white blood cell.

* Thirty‐two patients had measurable level of *bcr‐abl1* transcript after induction therapy.

** Forty patients, excluding three patients (two patients did not achieve CR, and one patient died within 3 months of achieving CR), were included for analysis.

### 2.2 Targeted next‐generation sequencing and somatic variant analyses

Next‐generation sequencing (NGS) was performed using bone marrow samples harvested from all 43 patients at their initial diagnosis. In addition, 37 CR samples (of 41 patients who achieved CR), 20 samples post‐transplantation (day 21) (of 25 patients who received allogeneic hematopoietic transplantation), and 15 relapse samples (of 16 patients who relapsed) were available and subject to NGS assessment. NK cell fractions using the BD FACSAria™III Cell Sorter (CD56‐Allophycocyanin [APC] [BD Biosciences, CA, USA]), were fractionated from the 43 diagnostic samples for use as the controls. Deep sequencing was performed by targeting the exonic coding regions of 45 genes with recurrent driver mutations, on the basis of prior data extracted from large cohort studies focused on ALL (Table S1) [25, 26, 27, 28, 29, 30]. To cover the genes related to clonal hematopoiesis and chromosomal arm‐level copy numbers, 1129 single nucleotide polymorphism sites were selected. Detailed procedures on sample preparation, NGS, and variant calling are provided in the Supplementary Materials. The mean on‐target coverage for 158 samples was 1130 × (interquartile range 1047–1230). We defined the threshold of mutation positivity in the samples obtained at diagnosis as a variant allele frequency (VAF) ≥ 3%. CNVPanelizer was used to assess CNAs in diagnosis, relapse, and follow‐up samples using CR samples as control [31]. We targeted the CNAs for 28 genes that are frequently reported in Ph‐positive ALL, and we set the cutoff value as a mean copy ratio of >1.15 or <0.85 [12, 32] (Table S2). The assessment of measurable residual disease was validated via RT‐PCR analysis for the levels of *BCR*‐*ABL1* transcripts using a Real‐Q *BCR*‐*ABL1* Quantification commercial Kit (BioSewoom, Seoul, Korea). A 5‐log reduction was defined as complete molecular remission (CMR), and a 3‐log reduction was defined as a major molecular response (MMR).

### 2.3 Statistical analyses

The survival analysis was performed following the protocol outlined in a previous study [33]. Consequently, Mantel–Byar tests were used to compare the survival data, the Simon and Makuch method was used for graphical representation, and time‐dependent multivariate Cox proportional hazard models were used to examine time‐dependent covariates by considering allogeneic HCT as a time‐dependent covariate [34]. All statistical analyses were performed using the EZR software, using the “R” language (available at http://www.jichi.ac.jp/saitama‐sct/SaitamaHP.files/statmedEN.html) [35].

## 3 RESULTS

### 3.1 Patient characteristics and impact of clinical factors

The median age was 46 years (range, 18–69 years) (Table 1). Twenty‐nine patients received hyper‐CVAD regimens with imatinib (*n* = 24) or dasatinib (*n* = 5), and 14 patients were administered the VPD regimen with imatinib (*n* = 10) or nilotinib (*n* = 4). Forty‐one (95%) of the 43 patients obtained morphologic CR after induction therapy. MMR after first induction chemotherapy was observed in 18 (56%) of the 32 patients for whom measurements were possible, and CMR was observed in 25 of 41 patients (58%) during chemotherapy and before the implementation of allogeneic HCT. The median interval from CR to allogeneic HCT was 3.0 months (range, 0.8–5.8 months) for the 25 patients (61%). Allogeneic HCT was performed in 25 patients (61%) of the 41 patients who had achieved CR. Transplantation could not be performed in the remaining 16 patients owing to disease progression (*n* = 4), nonavailability of a donor (*n* = 8), or patient refusal (*n* = 4). Of these, 14 patients (56%) received myeloablative conditioning regimens during allogeneic HCT. It was found that chronic graft versus host disease was present in 12 patients. The median follow‐up duration was 9.5 years (range, 0.4–10.9) among survivors; among those who had obtained CR, 16 patients (39%) showed relapse and 25 patients (61%) died.

The median duration from CR to relapse was 8.4 months (range, 2.3–41.4) for the 16 patients who had relapsed and two who were alive at their last follow‐up. Nine patients received salvage intensive chemotherapy with TKIs, and one was alive after donor lymphocyte infusion. Five patients received different types of TKIs only after relapse, and one of them was alive at the last follow‐up after the 2nd allogeneic HCT. One patient underwent a second salvage allogeneic HCT, and the other patient received conservative treatment only.

In the group that had reached CR (*n* = 41), the 5‐year OS, relapse‐free survival (RFS), cumulative incidence of relapse (CIR), and nonrelapse mortality (NRM) were 43.7% (95% CI, 27.7%–58.6%), 36.3% (95% CI, 21.6%–51.3%), 51.1% (95% CI, 34.1%–65.8%), and 12.5% (95% CI, 4.5%–25.0%), respectively. In the transplant group (*n* = 25), the 5‐year OS, RFS, CIR, and NRM were 65.7% (95% CI, 42.5%–81.3%), 58.4% (95% CI, 36.2%–75.2%), 24.0% (95% CI, 9.5%–42.1%), and 17.6% (95% CI, 5.2%–36.1%), respectively (Figure S1). A univariate analysis indicated that receiving allogeneic HCT was associated with significant prognostic improvements in OS, RFS, and CIR (all, *p* < 0.05) (Figure S1 and Table S3). When considering other clinical factors, white blood cell (WBC) counts at diagnosis had an impact on OS; the type of induction chemotherapy (VPD regimen) was associated with improved RFS; and poor performance status at diagnosis also increased the CIR (all, *p* < 0.05). The administration of 2nd‐generation TKIs was associated with an improved RFS (*p* = 0.067), and the achievement of CMR during chemotherapy tended to accompany a favorable prognosis, although without statistical significance (Figure S2, Table S3). Multivariate analysis revealed that allogeneic HCT was favorable for OS, RFS, and CIR (all, *p* < 0.05). The achievement of CMR during chemotherapy and the administration of 2nd‐generation TKIs significantly improved the RFS. The high WBC counts at diagnosis were associated with inferior OS, and the poor performance status at diagnosis also increased the CIR (all, *p* < 0.05) (Table 2). The benefits of allogeneic HCT were observed generally in Ph‐ALL, irrespective of other clinical factors and genetic alterations (Figure 3).

**TABLE 2:** Multivariate analysis of prognostic factors for nonrelapse mortality (NRM), cumulative incidence of relapse (CIR), relapse‐free survival (RFS), and overall survival (OS)

	OS		RFS		CIR		NRM	
	HR (95% CI)	*p*‐Value	HR (95% CI)	*p*‐Value	HR (95% CI)	*p*‐Value	HR (95% CI)	*p*‐Value
WBC, log scale	**1.908 (1.020–3.570)**	**0.043**						
Performance status at diagnosis (ECOG 0–1 vs. 2)					**2.903 (1.145–5.595)**	**0.025**	NA**	NA**
Type of induction chemotherapy (Hyper‐CVAD vs. VPD)	0.549 (0.187–1.616)	0.277	0.420 (0.151–1.165)	0.096				
Type of TKIs (imatinib vs. others)			**0.183 (0.049–0.676)**	**0.011**				
Achievement of CMR during chemotherapy (%)			**0.285 (0.121–0.673**)	**0.004**				
**Allogeneic HCT** *	**0.143 (0.054–0.383)**	**<0.001**	**0.150 (0.055–0.406)**	**<0.001**	**0.273 (0.101–0.735)**	**0.001**		

*Note*: Significant variables are shown in bold.

Abbreviations: CI, confidence interval; ECOG, Eastern Cooperative Oncology Group; HCT, hematopoietic cell transplantation; HR, hazard ratio; Hyper‐CVAD, cyclophosphamide, vincristine, doxorubicin, and dexamethasone; VPD, daunorubicin, vincristine, and prednisolone; WBC, white blood cell.

* Forty patients, excluding three patients (two patients did not achieve CR and one patient died within 3 months of achieving CR) were indicated for analysis.

** No event occurred in one group (ECOG 2 group, and group treated with 2nd line TKIs).

### 3.2 Molecular mutations and CNAs at diagnosis

**FIGURE 1:**
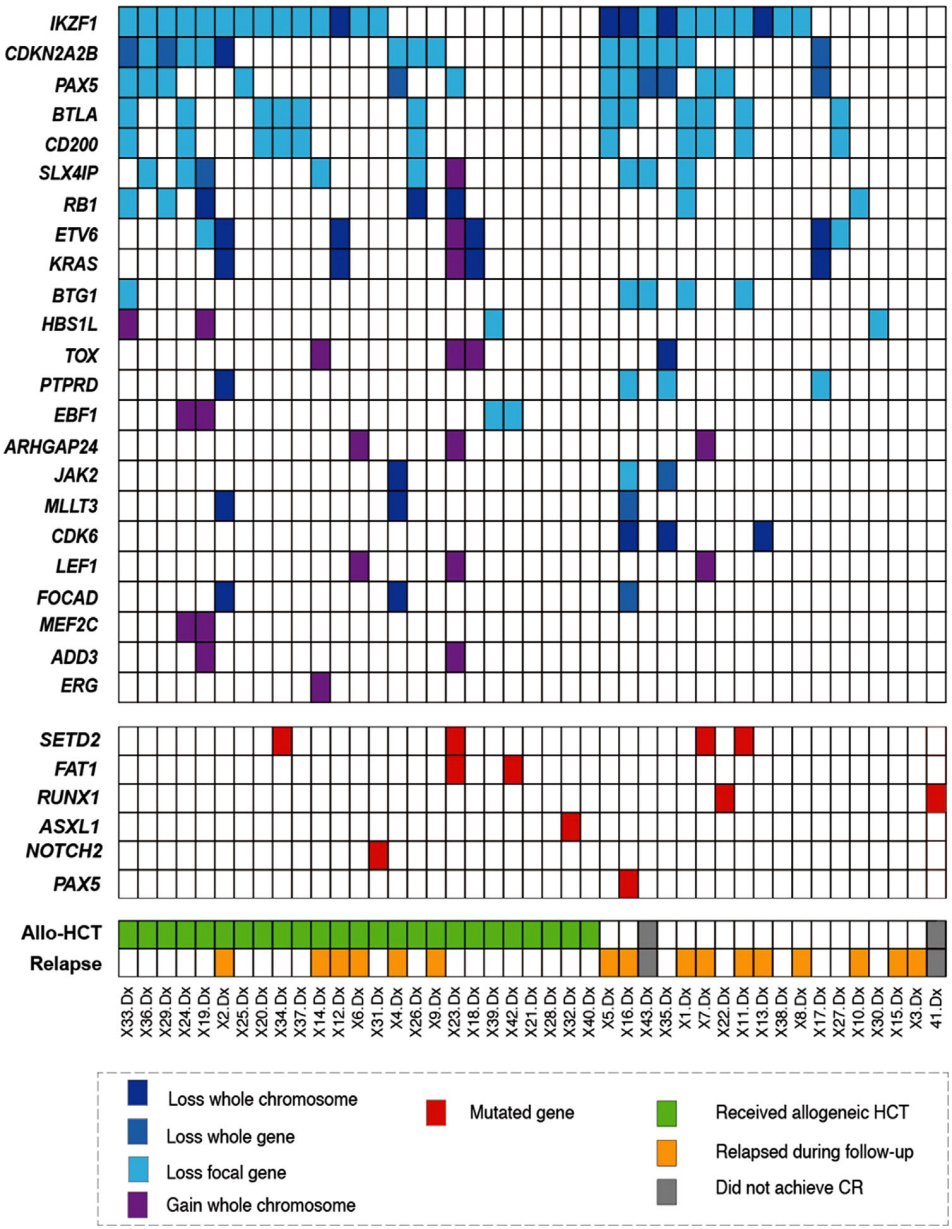
Prevalence of genetic alterations in the samples obtained at diagnosis from patients with Philadelphia‐positive acute lymphoblastic leukemia (*n* = 43)

In the samples obtained at diagnosis, we detected at least one genetic alteration in 38 (88%) of the 43 patients (Figure 1). The CNA analysis detected genetic aberrations in 36 patients (84%) from the samples collected at diagnosis. The most frequently detected CNA lesions were observed in *IKZF1* (*n* = 25, 58%) followed by *CDKN2A*/*B* (*n* = 15, 35%), *PAX5* (*n* = 13, 30%), *BTLA* (*n* = 12, 28%), and *CD200* (*n* = 11, 26%) (Figure 1). Of the 43 patients, a total of 10 (23%) harbored 12 mutations with a median VAF of 23.33% (range, 4.84%–46.67%) (Figure 1 and Table S4). The most frequently mutated gene was *SETD2* (*n* = 5) followed by *RUNX1* and *FAT1*(all, *n* = 2). Mutations in *ASXL1*, *NOTCH2*, and *PAX5* were observed in one patient each. One patient had two *SETD2* mutations and one harbored two (*FAT1* and *SETD2*) mutations (Figure 1).

Of the frequently detected CNAs found in the samples obtained at diagnosis, we could not determine the prognostic significance for any associated gene (Figure S2 and Table S3). Even *IKZF1* deletions, which were the most frequently detected CNAs, did not influence the treatment result in our cohort. The clinical significance of each genetic mutation could not be analyzed owing to the small number of patients that had each of the mutations. Further, there were no significant differences in the prognosis or in the OS, RFS, CIR, and NRM between the groups with a genetic mutation and the groups in which no mutations were detected (all, *p* > 0.05) (Figure S2).

### 3.3 Dynamics of genetic alterations at remission, post‐transplantation, and relapse

**FIGURE 2:**
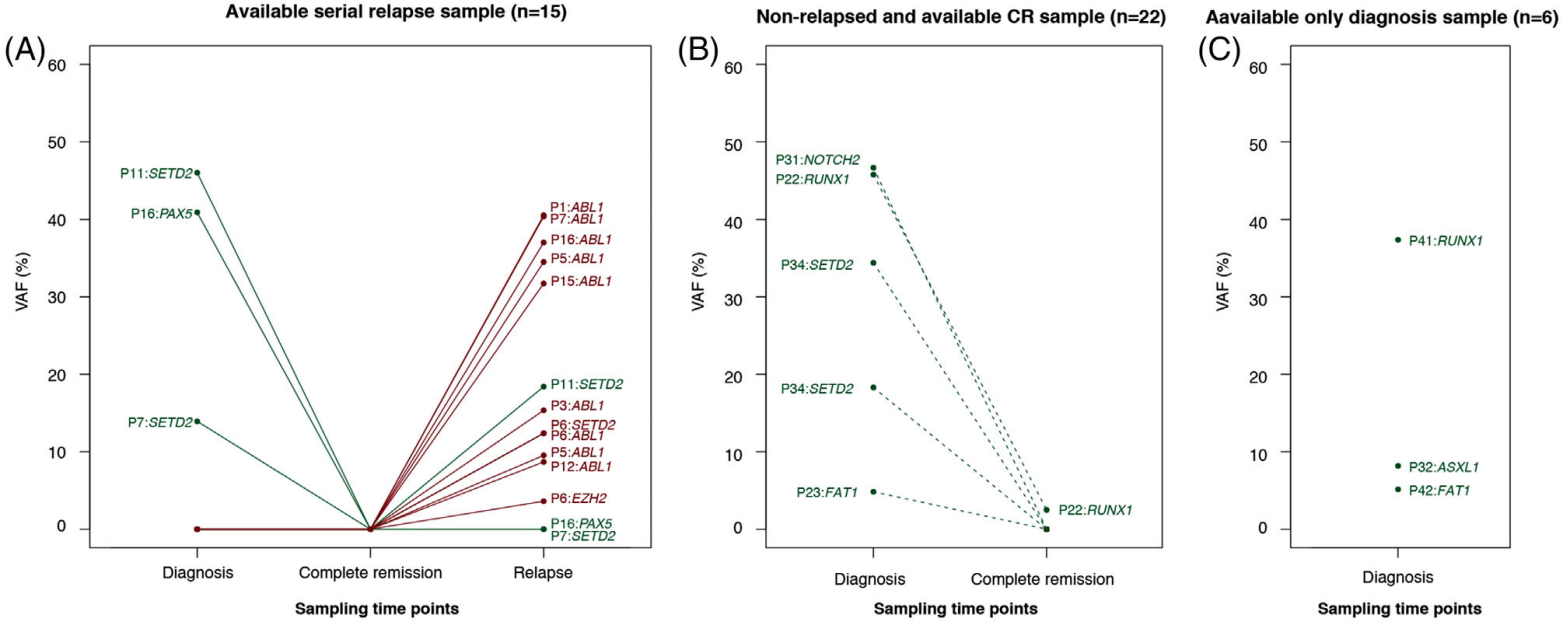
Dynamics of the variant allele frequencies (VAFs) of mutations at each time point. (A) Dynamics of VAFs in 15 relapsed patients available sequencing in three‐time points. (B) Dynamics of VAFs in 22 patients with available complete remission (CR) samples and who maintained a nonrelapse state. (C) VAFs at diagnosis in six patients, two patients did not achieved CR (P41, P43), and four patients were not available the samples at CR state (one relapsed [P14] and three nonrelapsed patients [P20, P32, P42]). Color means the mutations detected at diagnosis (green) or newly detected at relapse (red).

We sequentially analyzed the samples available from 37 patients showing CR and samples obtained on day 21 post‐transplantation from 20 patients. While screening for genetic alterations in the samples obtained at CR and the post‐transplantation stage, we did not find any genetic mutations or CNA lesions. When tracking the mutations observed at diagnosis, no mutations were detected, except those in *RUNX1*, which remained at a low VAF (2.5%) in the samples obtained after CR (Figure 2).

**FIGURE 3:**
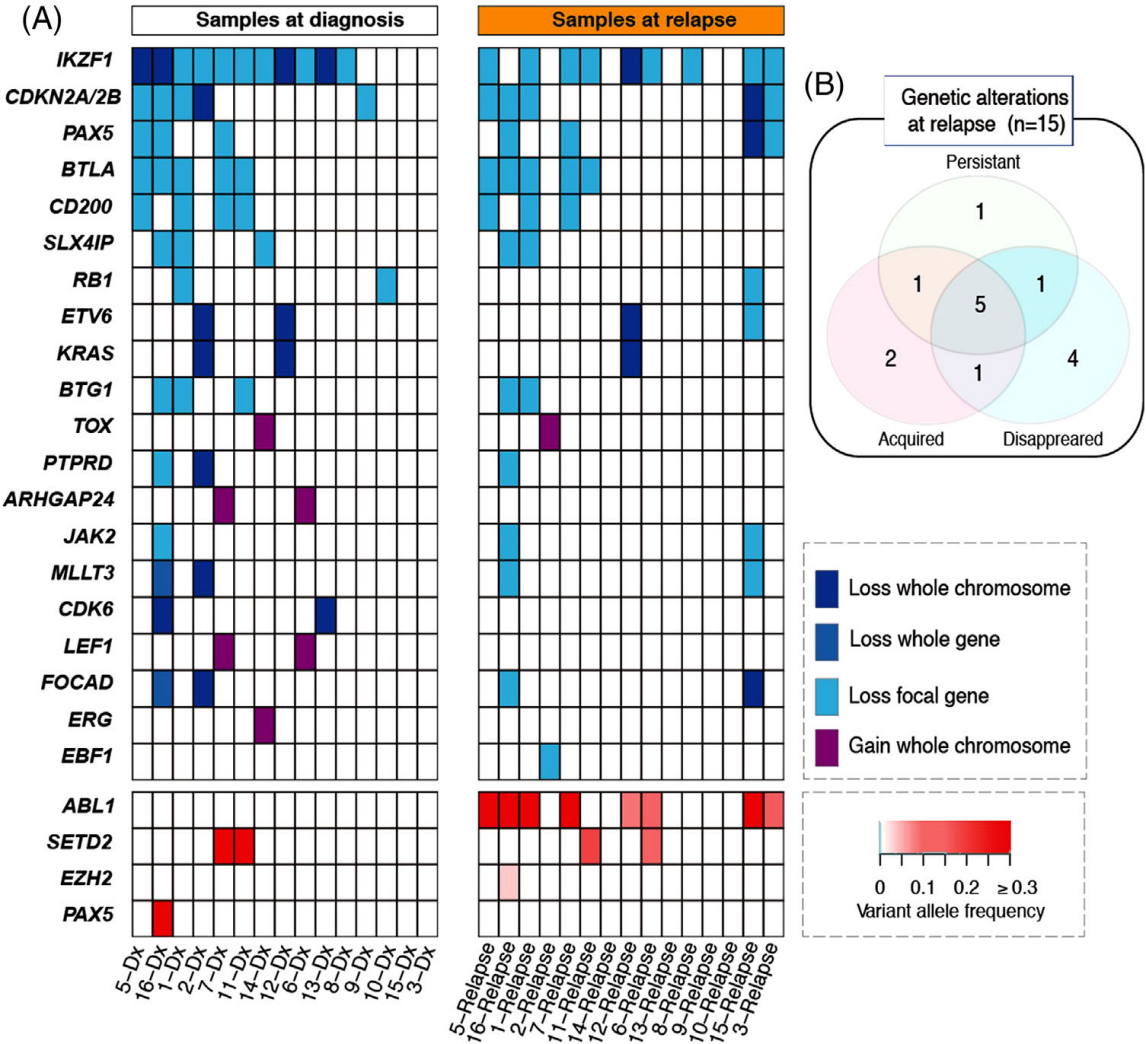
Prevalence of copy number aberrations and genetic mutations in paired samples obtained at diagnosis and relapse (*n* = 15). The Venn diagram shows the genetic changes at relapse compared with those at diagnosis.

In addition, we obtained samples from 15 of the 16 patients showing relapse; we analyzed these samples at the following time points: diagnosis, CR, and relapse. We detected the *BCR*‐*ABL1* transcript in all the patients at relapse. In addition, we detected at least one genetic abnormality (loss, gain, or persistence) in all the samples obtained at relapse. The CNA analysis discovered genetic aberrations at relapse in 11 patients (73%) (Figure 3A). The most frequently deleted CNA lesions were observed in *IKZF1* (*n* = 9, 60%) followed by *CDKN2A*/2*B* (*n* = 5, 33%), *BTLA* (*n* = 5, 33%), *CD200* (*n* = 5, 33%), and *PAX5* (*n* = 4, 27%). The *IKZF1* deletion, persistently detected in seven patients, disappeared in four patients. However, a new *IKZF1* deletion was identified in the samples from two patients at relapse. Twelve somatic mutations in nine patients (60%) were detected with a median VAF of 18.41% (range, 3.61%–40.53%) (Figures 2 and 3 and Table S4). The most frequently mutated gene was *ABL1* (eight patients, 53%), followed by *SETD2* (two patients, 5%), and *EZH2*(one patient) (Figure 2). Two patients harbored two (double *ABL1* mutations) or three (*ABL1*, *EZH2*, and *SETD2*) mutations. All somatic mutations were observed only at relapse, except for the mutation in *SETD2*. In one patient, the *SETD2* mutation was observed at the time of diagnosis (VAF, 46.02%); the frequency of this mutation was undetectable in the samples obtained at CR (VAF, 0%) and increased in the samples obtained at relapse (VAF, 18.41%) (Figure 2 and Table S4). The dynamics of the detected mutations according to the time points of analysis are depicted in Figure 2. Upon comparing the paired samples obtained at diagnosis and relapse, previously detected genetic alterations in the samples obtained at diagnosis disappeared in 11 patients (73%) and new genetic abnormalities that were not present at the time of diagnosis and CR were observed in nine patients (60%). Persistent genetic abnormalities were observed in eight patients (53%) (Figure 3B).

We traced back the serial samples to determine whether the *ABL1* mutations detected in the samples obtained at relapse could be detected in the samples collected at diagnosis. All *ABL1* mutations observed at relapse were not detectable at the time of diagnosis and at remission in the samples from the respective patients (Figure 2). When tracking the location of *ABL1* KD in all the samples, the *ABL1* KD mutation, with a low VAF, was detected in the nonrelapse samples from two patients, one patient at the time of diagnosis (Q252H; VAF, 0.98%), and another at CR (Q333K, VAF, 0.46%). However, these mutations were not clinically significant and disappeared during the treatment process.

## 4 DISCUSSION

We performed a sequential targeted DNA sequencing to identify genetic alterations during disease progression in adults with Ph‐ALL. Structure variations, especially, deletions, were more frequently observed in Ph‐ALL than genetic mutations at diagnosis. *ABL1* mutations were also frequently detected at relapse. Complex genetic alterations occurred in all patients with Ph‐ALL at relapse.

Genetic alterations (loss, gain, or persistence at relapse) were observed in all samples obtained at relapse, compared with the samples obtained at the time of diagnosis. Fourteen of 15 patients showed changes in genetic abnormalities, and only one patient showed persistent *IKZF1* deletion without any other genetic alterations. It can be inferred that the Ph‐positive leukemic clone progresses as a result of complex genetic changes involving the acquisition or loss of genetic alterations during the disease, and that genetic instability may lead to refractoriness to salvage treatment. Genetic mutations were not frequently detected in Ph‐ALL at diagnosis (detectable only in 23%) and rarely at relapse (detectable in 13%), except for *ABL1* KD mutations (*n* = 8/15, 53%). In only one patient, the *SETD2* mutation that was observed at diagnosis was undetectable in CR and increased at relapse. Ma et al. reported that a median of 18 mutations was observed at diagnosis through extensive analysis using deep whole‐exome sequencing in children with B‐ALL [8]. In other sequencing results using the targeted exons of 950 genes related to cancer, the number of mutations per patient in Ph‐ALL was reported to be as low as 0.8 (range, 0–2) [9]. The most frequently mutated genes were found to be members of the RAS signaling pathway, and *RAS* mutations are known to be more abundant in B‐ALL [9]. In contrast, we were unable to define the molecular mutations commonly found in Ph‐ALL at diagnosis. Only 23% of the patients carried one or two other mutations at diagnosis in our study. This is probably because, despite deep sequencing, only 45 genes (exonic regions only) were targeted in our study. It is considered that mutations were observed at a low frequency because extensive exonic lesions were not included in our panel. One of the reasons for this observation may be that adult patients with Ph‐ALL are reported to have significantly lower frequencies of alterations in the RAS pathway‐associated genes than the patients with B‐ALL [26].

As reported in other studies, *IKZF1* or *CDKN2A/2B* were frequently observed at diagnosis in our cohort [12, 20, 26]. These results support the hypothesis that *BCR‐ABL1*, combined with the deletion of *IKZF1* or *CDKN2A/2B*, participates in the development of Ph‐ALL [20]. The most frequently observed deletions in the samples obtained at relapse were seen in *IKZF1* (*n* = 9, 60%). This was persistently detected in seven patients, and a new *IKZF1* deletion was identified in the samples obtained from two patients at relapse. Alterations of *IKZF1* play a fundamental role in Ph‐ALL [36].

To drive B‐lymphoid development, Ikaros has been reported to repress hematopoietic stem‐cell‐specific gene expression programs during early lineage specification [37, 38]. Additionally, *IKZF1* deletion in B‐ALL shows a more stem cell‐like signature in gene expression profiling than the wild‐type *IKZF1*, and the expression of a stem cell program has been associated with drug resistance and poor outcomes in other types of leukemia [39, 40, 41]. The relapse pattern of our study suggests a role for *IKZF1* in mediating drug resistance and relapse.

Consistent with previous reports, the most significant mutations at relapse were the *ABL1* mutations; these were observed in 53% of the samples obtained at relapse in our study [42, 43]. Interestingly, we could not find concordance with regard to *ABL1* mutations in the serial samples at the time of diagnosis, remission, and relapse even during the tracking of the detected *ABL1* mutations. In several studies, *ABL1* mutations were observed with extremely low allelic burdens in a substantial proportion of patients with Ph‐ALL, eventually leading to an enhanced risk of relapse [42, 43]. Schmitt et al. reported that all *ABL1* mutations observed at relapse were detected at diagnosis by deep sequencing, but that mutations could not be detected by NGS or sanger sequencing in 93% of the same samples obtained at diagnosis [43]. Therefore, our results suggest that clinically applicable NGS panels are limited in their ability to detect the presence of *ABL1* mutations at diagnosis. Further studies are needed to elucidate the concordance of preexisting subclones with clinically actionable resistant clones in order to be able to choose TKIs according to the mutation profile at diagnosis [44]. It is reported that the survival rate is significantly improved by reducing the risk of relapse when 2nd‐ and 3rd‐generation TKIs are used in combination with chemotherapy and maintenance therapy; this may represent one observation that supports the aforementioned findings [4, 7, 45]. Based on the mutation analysis at the time of relapse, it can be interpreted that the use of potent TKIs that can overcome the resistance caused by *ABL1* mutations is helpful in preventing relapse.

We demonstrated the dynamics of genetic alterations via the analyses of serial samples of Ph‐ALL using an NGS panel that can be easily applied in clinical practice. Since the prognosis in Ph‐ALL is extremely poor owing to complex genetic alterations at the time of relapse, it is suggested that the incidence of relapse may be reduced through the use of potent TKIs and allogeneic HCT in these patients. Our results support the need for the further evaluation of relapse patterns associated with the use of more potent TKIs and other novel agents, so as to introduce more effective treatments for preventing relapse in Ph‐ALL patients.

## AUTHOR CONTRIBUTIONS

JSA, HJK, and DK designed the study. JSA, SHJ, SYA, GYS, MK, DHY, JJL, SHC, MYK, and HJK collected the samples and performed the experiments. JSA, THK, and ZZ analyzed the sequencing data. JSA, THK, JHM, and DK interpreted the data and performed statistical analyses. JSA, THK, HJK, ZZ, and DK wrote the paper. JSA and THK contributed equally to this work.

## CONFLICT OF INTEREST

The authors declare no competing financial interests.

## Supporting information

Supplementary information

Supplementary information

## Data Availability

All sequencing data have been deposited at the European Nucleotide Archive (accession number: PRJEB48507) https://www.ebi.ac.uk/ena/browser/view/PRJEB48507?show=reads.
