# Supplementary material for: Next‐generation sequencing‐based analysis to assess the pattern of relapse in patients with Philadelphia‐positive acute lymphoblastic leukemia
Source: EJHaem. 2022 Sep 9;3(4):1145–53. doi: 10.1002/jha2.514 (PMC9713221; doi:10.1002/jha2.514)
Supplement: Supplementary file 1 — Supplementary information [file JHA2-3-1145-s001.docx]

**Next-generation sequencing-based analysis to assess the pattern of relapse in patients with Philadelphia-positive acute lymphoblastic leukemia**

Jae-Sook Ahn*_1,2,_ TaeHyung Kim*_3, 4_, Sung-Hoon Jung*_1_, Seo-Yeon Ahn_1_, Ga-Young Song_1_, Mihee Kim_1_, Deok-Hwan Yang_1_, Je-Jung Lee_1_, Mi Yeon Kim_2_, Joon Ho Moon_5_, Zhaolei Zhang‡_3,4,6_ Hyeoung-Joon Kim‡_1,2_, and Dennis Dong Hwan Kim‡_7_

_1_ Department of Internal Medicine, Chonnam National University Hwasun Hospital, Chonnam National University, Gwangju, Republic of Korea

_2_ Genomic Research Center for Hematopoietic Diseases, Chonnam National University Hwasun Hospital, Jeollanam-do, Republic of Korea,

_3_ The Donnelly Centre for Cellular and Biomolecular Research, University of Toronto, Toronto, ON, Canada

_4_ Department of Computer Science, University of Toronto, Toronto, ON, Canada

_5_Department of Hematology-Oncology, Kyungpook National University Hospital, School of Medicine, Kyungpook National University, Daegu, Republic of Korea

_6_Department of Medical Oncology and Hematology, Princess Margaret Cancer Centre, University of Toronto, Toronto, Canada

_7_ Department of Molecular Genetics, University of Toronto, Toronto, ON, Canada

**Materials**

***Patients***

Twenty-nine patients received the hyper-CVAD induction therapy [cyclophosphamide 300 mg/m^2^ on days 1–3, vincristine 1.4 mg/m^2^ on days 3 and 11, doxorubicin 50 mg/m^2^ on day 3 and dexamethasone 40 mg on days 1–4 and 11–14 (cycle A)] with imatinib mesylate 600 mg (n=24) or dasatinib 140 mg (n=5). This was then followed 21 days later by methotrexate 1 gm/m^2^ on day 1, ara-c 3 gm/m^2^ on days 2–3, and methylprednisolone 100 mg on days 1–3 (cycle B) [1]. The other 14 patients were administered VPD regimen [daunorubicin 90 mg/m^2^ on days 1**–**3, vincristine 2 mg on days 1 and 8, and oral prednisolone 60 mg/m^2^ on days 1–14 for remission induction. Bone marrow was examined on day 14 and patients with residual leukemic blasts received an additional dose of daunorubicin 45 mg/m^2^] with imatinib 600 mg (n=10) or nilotinib 800 mg (n=4) [2, 3]. All procedures followed were in accordance with the ethical standards of the responsible committee on human experimentation (institutional and national) and with the Helsinki Declaration of 1975, as revised in 2008. Informed consent was obtained from all patients for being included in the study.

***Sample preparation and next-generation sequencing***

After library preparation, all samples were subjected to targeted sequencing per the manufacturer’s instructions. Using the Agilent (SureSelect Target Enrichment) custom probe set targeting the coding region of 45 genes and 1,121 single nucleotide polymorphism (SNP) sites (Santa Clara, California, USA), all samples were sequenced using an Illumina Hiseq 2500 (San Diego, California, USA) with a 101-bp pair-end mode. Sequencing metrics of 158 samples (43 samples at diagnosis, 43 samples with NK cells for control, 37 samples at diagnosis, 20 samples after transplantation, and 15 samples at relapse) for targeted sequencing.

***Next-generation sequencing read processing and procedure for variant calling***

Each sequencing read was processed using the methodology described in our previous study [8]. Each read was first mapped by the human genome (hg19) using Burrow–Wheeler Aligner [9]. After marking PCR duplicates using the Picard toolkit [10], we realigned insertions and deletions, fixed mate information, and recalibrated the base score using the Genome Analysis toolkit [11]. We discarded reads with more than one mismatch, counting insertions and deletions as one mismatch regardless of size to reduce background error rates [12]. During the variant calling procedure, samples of NK cells were used as controls. Our initial variant calling procedures were as follows:

1. Variants deemed significant in the currently considered case by Fisher’s exact tests on allele counts in diagnosis and their corresponding NK cell samples.
2. In addition to *p* ≤ 0.005 from Fisher’s exact test, we require these criteria to be fulfilled for further consideration:
3. Variant allele frequency (VAF) in diagnostic samples ≥ 3%
4. VAFs in NK cell samples < 5%
5. VAFs in diagnostic samples > VAFs in NK cell samples
6. Minimum coverage of 30×
7. Presence of reads on both forwards and reverse strands in diagnostic samples

Next, we backtracked, searching for occurrences of these variants in all diagnostic samples as the significance of a variant in any sample from our cohort increases the probability of it being a real somatic mutation in other samples. Finally, we tracked identified mutations in corresponding NK cell samples. For all mutations, we required VAFs to be a minimum of 3% in diagnostic samples. For recurrent mutations that were identified in multiple cases, we searched for variants in which the control sample VAF was greater than a third of the tumor sample VAF. We removed these unless they occurred in a minimum of 25 cases in the Catalog of Somatic Mutations In Cancer (COSMIC) database (hematopoietic and lymphoid tissues only) [14]. For each of these variants, we counted the total number of cases that met this condition as well. If this count reached 10% of the number of cases with control samples, the variant was removed from all cases (including occurrences that did not meet this condition). For variants that did not occur in a minimum of five hematopoietic and lymphoid tissue cases in the COSMIC database, if they occurred in any case with a germline VAF greater than a third of the tumor VAF, they were removed from all cases, regardless of the number of times they occurred. All sequencing data have been deposited at the European Nucleotide Archive (Accession number: PRJEB48507).

For the tracking *ABL1* KD mutations in every time-series samples, we extracted the exonic lesion of *ABL1* kinase domain from samples, and selected the variants with two or more read counts reported as mutations in Catalogue of Somatic Mutations in Cancer data. We used Genome Analysis ToolKit4 (GATK4) to detect copy number aberrations (CNA) for diagnosis and matched NK cell controls [4]. We followed the best practices workflow of somatic copy number variant discovery (CNAs). To build the Panel of Normals (PON), we used 43 sample of NK cell control. We further analyzed the SNP sites included in mutation list to supplement the missing position in the sparse SNP selection.

**TABLE S1** Mutated gene list included in panel

**TABLE S2** Gene list for copy number alteration

**TABLE S3** Univariate analysis of prognostic factors for overall survival (OS), relapse-free survival (RFS), cumulative incidence of relapse (CIR), and non-relapse mortality (NRM)

|  | OS | | RFS | | CIR | | NRM | |
| --- | --- | --- | --- | --- | --- | --- | --- | --- |
|  | HR (95% CI) | *p-*value | HR (95% CI) | *p-*value | HR (95% CI) | *p-*value | HR (95% CI) | *p-*value |
| Age, decades | 1.329 (0.940-1.879) | 0.108 | 1.256 (0.901–1.750) | 0.179 | 1.123 (0.728–1.735) | 0.600 | 1.123 (0.729–1.730) | 0.600 |
| WBC, log scale | **2.038 (1.039-3.994)** | **0.038** | 1.496 (0.806–2.777) | 0.202 | 1.240 (0.625–2.460) | 0.540 | 1.425 (0.546–3.718) | 0.470 |
| Sex, male | 1.105 (0.501-2.439) | 0.804 | 1.078 (0.503–2.307) | 0.847 | 0.743 (0.270–2.048) | 0.570 | 2.041 (0.621–6.711) | 0.240 |
| Performance status at diagnosis (ECOG 0–1 vs. 2) | 1.055 (0.439-2.533) | 0.905 | 1.351 (0.588–3.103) | 0.479 | **3.612 (1.422–9.177)** | **0.007** | NA*** | NA*** |
| Type of induction chemotherapy  (Hyper-CVAD vs. VPD) | 0.410 (0.162-1.040) | 0.061 | **1.368 (0.144–0.937)** | **0.036** | 0.418 (0.118–1.484) | 0.180 | 0.640 (0.182–2.252) | 0.490 |
| Achievement of MMR after induction chemotherapy* | 1.336(0.504-3.703) | 0.539 | 1.233 (0.483-3.143) | 0.662 | 0.913(0.291-2.867) | 0.880 | 1.909(0.378-9.641) | 0.430 |
| Achievement of CMR during chemotherapy | 0.702 (0.313-1.573) | 0.389 | 0.506 (0.235-1.092) | 0.083 | 0.500 (0.192-1.305) | 0.160 | 1.040 (0.307-3.528) | 0.950 |
| Type of TKIs  (imatinib vs. others) | 0.470 (0.161-1.367) | 0.166 | 1.323 (0.097–1.080) | 0.067 | 0.489 (0.116–2.068) | 0.330 | 0.340 (0.044-2.648) | 0.300 |
| **Allogeneic HCT**** | **0.138 (0.056-0.341)** | **<0.001** | **0.176 (0.067–0.464)** | **<0.001** | **0.238 (0.089–0.634)** | **0.004** | 0.728 (0.205–2.592) | 0.620 |
| *IKZF1*^del^ | 1.077 (0.488-2.377) | 0.854 | 1.252 (0.579-2.706) | 0.568 | 1.926 (0.696-5.343) | 0.210 | 1.396 (0.599-3.253) | 0.440 |
| *CDKN2A/2B*^del^ | 1.02 (0.448-2.322) | 0.962 | 1.209 (0.551-2.655) | 0.637 | 1.237(0.452-3.387) | 0.680 | 0.810 (0.302-2.169) | 0.680 |
| *PAX5*^del^ | 1.478 (0.636-3.435) | 0.364 | 1.207 (0.528-2.760) | 0.656 | 0.596 (0.163-2.181) | 0.430 | 0.599 (0.199-1.800) | 0.360 |
| *BTLA*^del^ | 1.476 (0.650-3.35) | 0.352 | 1.183 (0.530-2.638) | 0.632 | 1.533 (0.573-4.103) | 0.400 | 1.364 (0.555-3.350) | 0.500 |
| *CD200*^del^ | 1.280 (0.551-2.973) | 0.565 | 1.035 (0.452-2.369) | 0.935 | 1.199 (0.434-3.310) | 0.730 | 1.121 (0.445-2.819) | 0.810 |
| *SLX4IP*^del^ | 1.135 (0.452-2.853) | 0.787 | 1.012 (0.407-2.512) | 0.980 | 1.480 (0.488-4.488) | 0.490 | 1.428 (0.547-3.728) | 0.470 |
| *RB1*^del^ | 0.609 (0.208-1.785) | 0.366 | 0.582 (0.201-1.689) | 0.320 | 0.962 (0.314-2.948) | 0.950 | 1.011 (0.394-2.593) | 0.980 |
| *ETV6*^del^ | 0.878 (0.300-2.567) | 0.812 | 1.121 (0.424-2.964) | 0.818 | 0.646 (0.143-2.910) | 0.570 | 1.103 (0.413-2.946) | 0.850 |
| *KRAS*^del^ | 1.119 (0.332-3.775) | 0.857 | 1.612 (0.554-4.691) | 0.381 | 1.069 (0.237-4.831) | 0.930 | 1.846 (0.397-8.624) | 0.430 |
| Any mutations | 1.217 (0.484-3.060) | 0.677 | 0.975(0.391-2.427) | 0.956 | 0.939 (0.262-3.364) | 0.920 | 1.361 (0.410-4.513) | 0.610 |

HR, hazard ratio; CI, confidence interval; WBC, white blood cell; ECOG, Eastern Cooperative Oncology Group; HyperCVAD, cyclophosphamide, vincristine, doxorubicin, and dexamethasone; VPD, daunorubicin, vincristine, and oral prednisolone; MMR, major molecular response; CMR, complete molecular response; TKIs, tyrosine kinase inhibitors; HCT, hematopoietic cell transplantation.

Significant variables are shown in bold.

*Thirty-two patients were available for analysis.

** Forty patients, excluding 3 patients [2 patients did not achieve complete remission (CR); 1 patient died within 3 months of achieving CR] were indicated for analysis.

*** No event occurred in ECOG 2 group.

**TABLE S4** Detailed description of all mutations discovered on the basis of targeted sequencing.

| Name of the column | Explanation |
| --- | --- |
| Sample ID | Sample ID |
| Chrom | Chromosome of mutation |
| position | Position of variants |
| Ref | Reference allele |
| var | variant allele |
| tumor_reads1 | reads supporting reference allele |
| tumor_reads2 | reads supporting variant allele |
| tumor_var_freq | Variant allele frequency |
| tumor_reads1_plus | Tumor reference-supporting reads on + strand |
| tumor_reads1_minus | Tumor reference-supporting reads on - strand |
| tumor_reads2_plus | Tumor variant-supporting reads on + strand |
| tumor_reads2_minus | Tumor variant-supporting reads on - strand |
| Func.refGene | Mutation type |
| Gene.refGene | Hugo gene nomenclature committee symbol |
| ExonicFunc.refGene | Mutation type |
| AAChange.refGene | Amino acid change |
| cytoBand | Chromosome band |

**FIGURE S1** Overall survival (OS), relapse-free survival (RFS), cumulative incidence of relapse (CIR), and non-relapse mortality (NRM) depending on whether allogeneic hematopoietic cell transplantation (HCT) was performed in patients with Philadelphia-positive acute lymphoblastic leukemia. The effects of allogeneic HCT at first complete remission on OS (**A**) and RFS (**B**) are represented as Simon–Makuch plots. (**C**) represents the CIR and (**D**) represents the cumulative incidence of NRM.

**
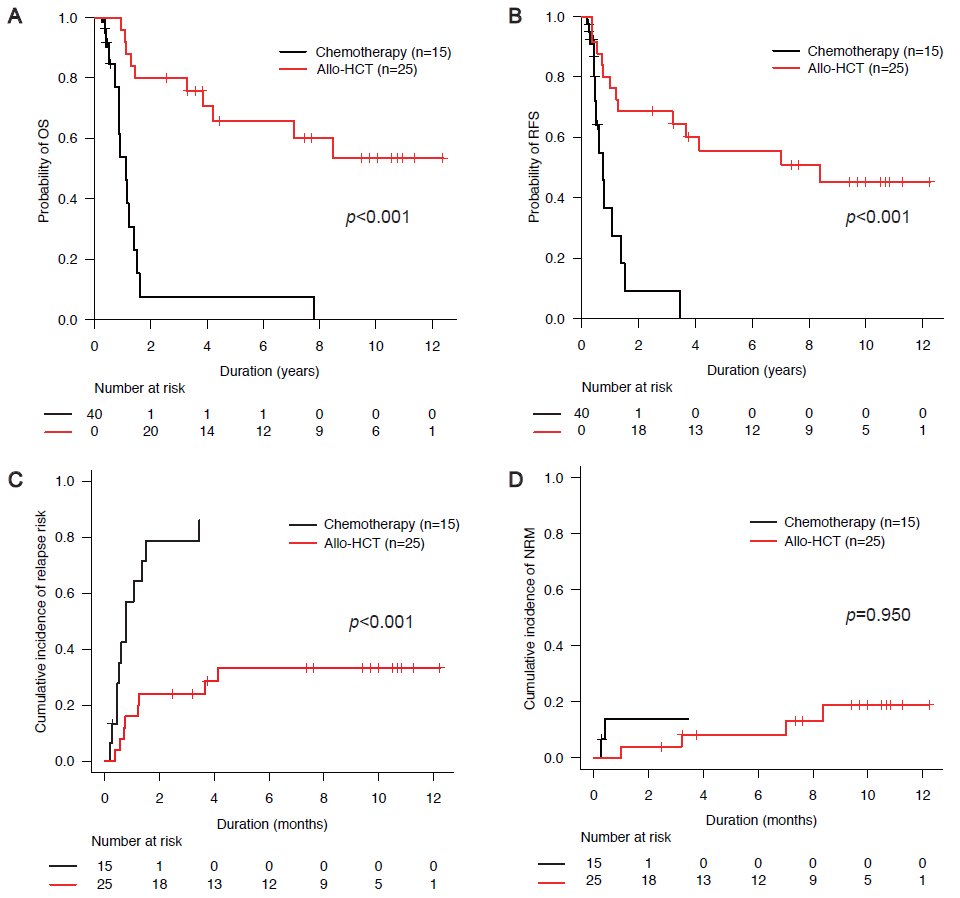
**

**FIGURE S2** Survival, relapse risks, and non-relapse mortalities according to clinical and genetic factors. Forest plots show the hazard ratios (HRs, solid square) with 95% confidence interval (blue line) for each variable. Significant variables are shown in bold. HR, hazard ratio; TKIs, tyrosine kinase inhibitors; MMR, major molecular response; CMR, complete molecular response; HCT, hematopoietic cell transplantation; NRM, non-relapse mortality. Variables denoted in an asterisk (*) mean factors that are not available the statistical analysis.

**
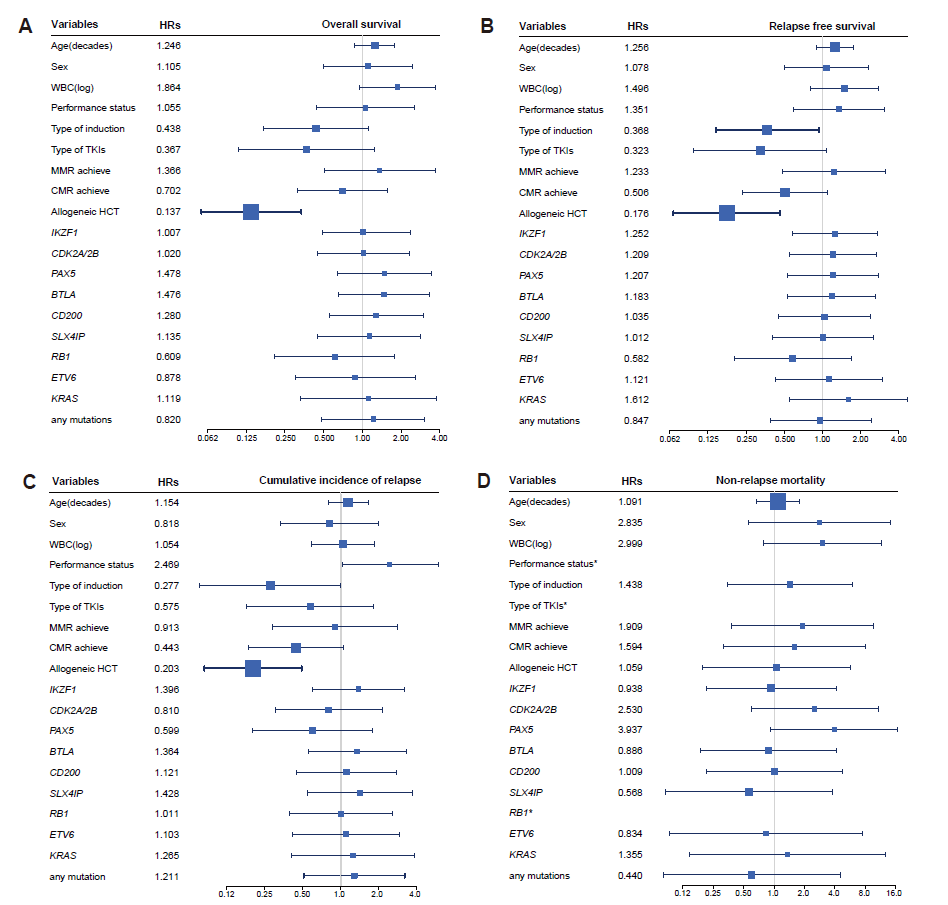
**

**FIGURE S3** Overall survival(A), relapse free survival(B), cumulative incidence of relapse risks (C), and non-relapse mortalities (D) according to the consolidation therapy [chemotherapy vs allogeneic hematopoietic cell transplantation (HCT)]. Forest plots show the hazard ratios (HRs, the solid square) with 95% confidence interval (the blue line) for each variable. Significant variables are shown in bold. HR, hazard ratio; TKIs, tyrosine kinase inhibitors; MMR, major molecular response; CMR, complete molecular response; NRM, non-relapse mortality; allo-HCT, allogeneic hematopoietic cell transplantation.


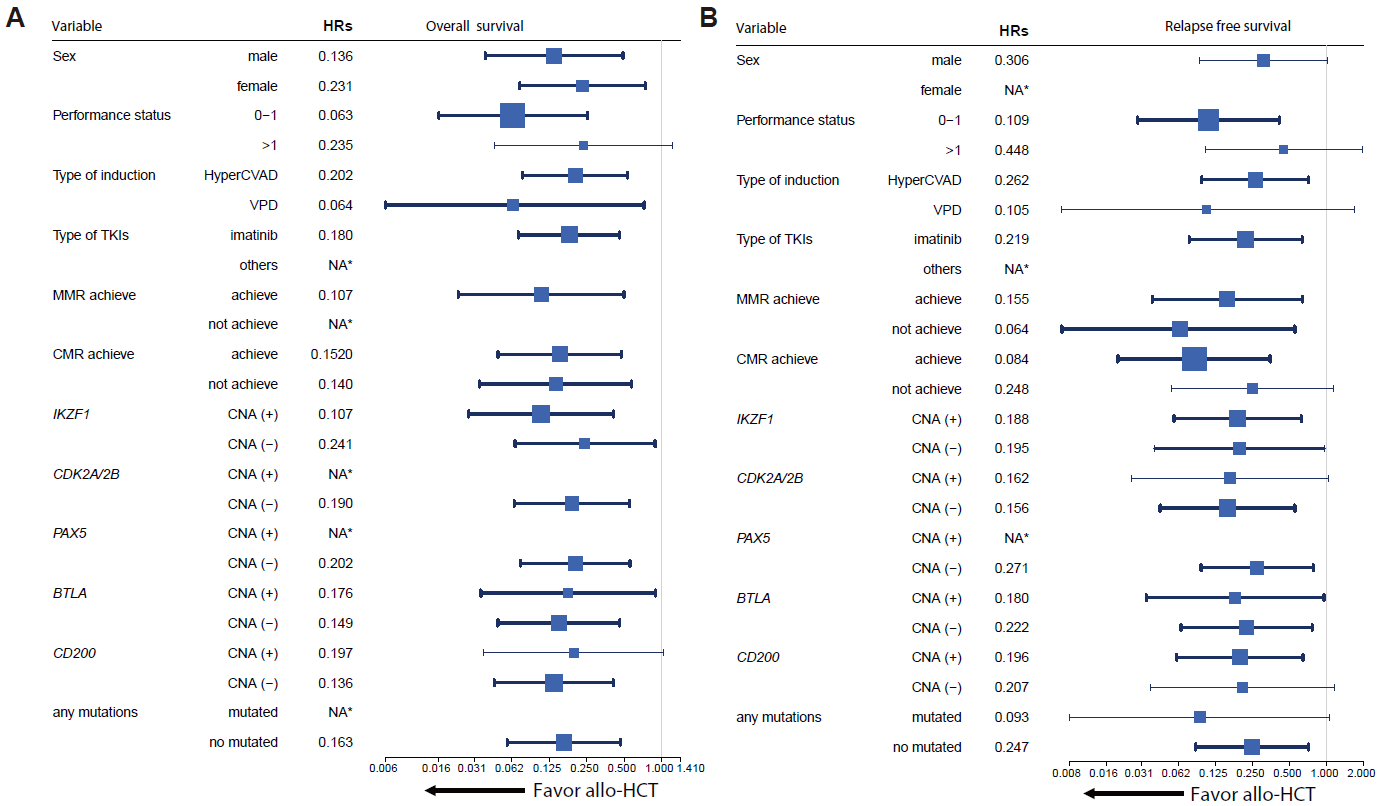

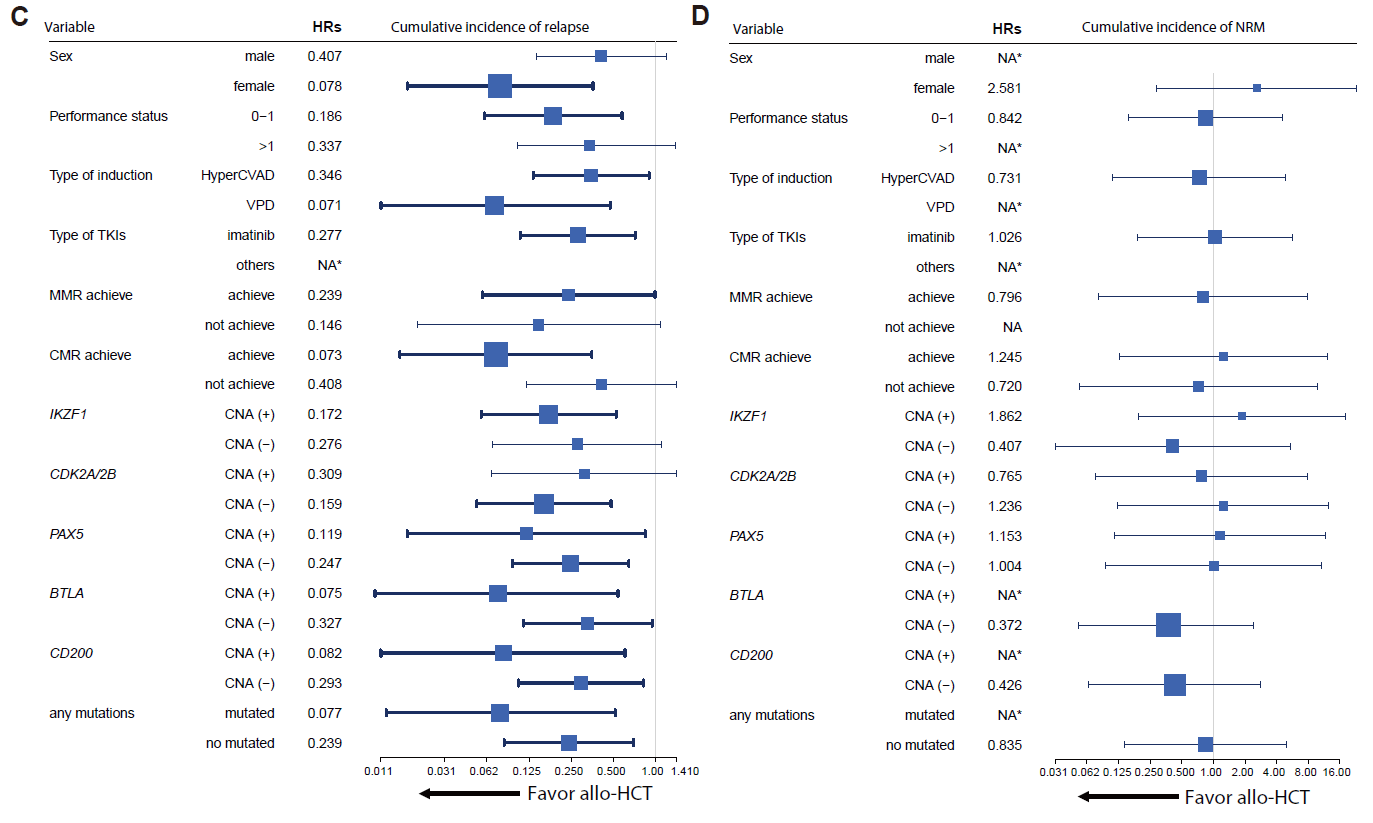


**REFERENCES**

1. Kantarjian HM, O'Brien S, Smith TL, Cortes J, Giles FJ, Beran M, et al. Results of treatment with hyper-CVAD, a dose-intensive regimen, in adult acute lymphocytic leukemia. J Clin Oncol. 2000;18(3):547-61.

2. Lim SN, Joo YD, Lee KH, Kim DY, Lee JH, Lee JH, et al. Long-term follow-up of imatinib plus combination chemotherapy in patients with newly diagnosed Philadelphia chromosome-positive acute lymphoblastic leukemia. Am J Hematol. 2015;90(11):1013-20.

3. Kim DY, Joo YD, Lim SN, Kim SD, Lee JH, Lee JH, et al. Nilotinib combined with multiagent chemotherapy for newly diagnosed Philadelphia-positive acute lymphoblastic leukemia. Blood. 2015;126(6):746-56.

4. Auwera Gvd, O'Connor BD. Genomics in the cloud : using Docker, GATK and WDL in Terra. First edition. ed. Beijing: O'Reilly; 2020. xxiv, 467 pages p.
